# The Onecut Transcription Factors Regulate Differentiation and Distribution of Dorsal Interneurons during Spinal Cord Development

**DOI:** 10.3389/fnmol.2017.00157

**Published:** 2017-05-26

**Authors:** Karolina U. Kabayiza, Gauhar Masgutova, Audrey Harris, Vincent Rucchin, Benvenuto Jacob, Frédéric Clotman

**Affiliations:** ^1^Université catholique de Louvain, Institute of Neuroscience, Laboratory of Neural DifferentiationBrussels, Belgium; ^2^Biology Department, School of Science, College of Science and Technology, University of RwandaButare, Rwanda; ^3^Université catholique de Louvain, Institute of Neuroscience, System and Cognition DivisionBrussels, Belgium

**Keywords:** onecut, transcription factors, spinal cord, dorsal interneurons, neural differentiation, neuronal migration, embryonic development

## Abstract

During embryonic development, the dorsal spinal cord generates numerous interneuron populations eventually involved in motor circuits or in sensory networks that integrate and transmit sensory inputs from the periphery. The molecular mechanisms that regulate the specification of these multiple dorsal neuronal populations have been extensively characterized. In contrast, the factors that contribute to their diversification into smaller specialized subsets and those that control the specific distribution of each population in the developing spinal cord remain unknown. Here, we demonstrate that the Onecut transcription factors, namely Hepatocyte Nuclear Factor-6 (HNF-6) (or OC-1), OC-2 and OC-3, regulate the diversification and the distribution of spinal dorsal interneuron (dINs). Onecut proteins are dynamically and differentially distributed in spinal dINs during differentiation and migration. Analyzes of mutant embryos devoid of Onecut factors in the developing spinal cord evidenced a requirement in Onecut proteins for proper production of a specific subset of dI5 interneurons. In addition, the distribution of dI3, dI5 and dI6 interneuron populations was altered. Hence, Onecut transcription factors control genetic programs that contribute to the regulation of spinal dIN diversification and distribution during embryonic development.

## Introduction

During spinal cord development, numerous neuronal populations that progressively subdivide into smaller and discrete subsets are generated in a space- and time-regulated manner from a collection of distinct progenitor domains orderly distributed along the ventro-dorsal axis of the neural tube (Lewis, 2006; Lu et al., 2015). Each population (Grossmann et al., 2010), and each subset it generates (Bikoff et al., 2016), migrates along stereotyped pathways and settles at well-defined locations in the developing spinal cord. Several observations suggest that the position of spinal neurons is a critical determinant of microcircuit organization. Indeed, the clustering and dorsoventral settling position of motor neuron pools serve as a determinant of the pattern of sensory input specificity (Sürmeli et al., 2011). Similarly, position of dorsal interneuron (dINs) along the mediolateral axis in lamina V determines their connectivity with sensory afferents (Ladle et al., 2007; Tripodi et al., 2011; Hilde et al., 2016). Furthermore, positional distinctions among V1 interneuron subsets (Bikoff et al., 2016) or Lbx1-derived premotor interneurons (Goetz et al., 2015) constrain patterns of input from sensory and motor neurons. Finally, multiple interneuron subsets are differentially distributed along the antero-posterior axis of the spinal cord, consistent with their integration into specific but distinct local microcircuits (Francius et al., 2013). The genetic programs that support the generation and the diversification of spinal interneurons have been extensively investigated (Lu et al., 2015). However, in contrast to cortical interneurons whose migration mechanisms have been largely deciphered (Guo and Anton, 2014; Barber and Pierani, 2016), the molecular determinants that regulate the distribution of interneuron populations in the developing spinal cord remain elusive.

Spinal interneurons generated from dorsal progenitor domains of the neural tube integrate and relay proprioceptive, mechanosensory or nociceptive information to motor circuits or to higher brain centers (Windhorst, 2007; Todd, 2010; Abraira and Ginty, 2013; Bui et al., 2015; West et al., 2015; Lai et al., 2016). Dorso-ventral (DV) gradients of BMP and Wnt signaling contribute to specify three dorsal progenitor domains (pdI1–3) according to their position along the DV axis (Wine-Lee et al., 2004; Timmer et al., 2005; Zechner et al., 2007; Tozer et al., 2013), whereas generation of the three more ventral domains (pdI4–6) occurs independently of these signals (Zhuang and Sockanathan, 2006; Le Dréau and Martí, 2012; Lai et al., 2016). Specific combinations of pro-neural factors and of bHLH or homeodomain proteins direct the identity of the post-mitotic neuronal populations generated from these domains (Zhuang and Sockanathan, 2006; Lai et al., 2016). Subsequently, dorsal cells acquire an inhibitory or excitatory phenotype through a complex integrated network of factors (Cheng et al., 2004, 2005; Mizuguchi et al., 2006; Henke et al., 2009; Chang et al., 2013; Borromeo et al., 2014; Hanotel et al., 2014). In contrast to spinal ventral interneurons, for which numerous subpopulations and subsets have been identified (Francius et al., 2013; Lu et al., 2015) and partly functionally characterized (Gosgnach et al., 2006; Zagoraiou et al., 2009; Borowska et al., 2013; Dougherty et al., 2013; Talpalar et al., 2013), the subdivision of dIN populations into more discrete subsets has only been sparsely investigated (Ding et al., 2004; Andersson et al., 2012; Dougherty et al., 2013; Bourane et al., 2015; Hilde et al., 2016). In addition, although these cells migrate according to highly specific and distinct directions and patterns, and display various final settling positions ranging from ventral to the dorsal most regions of the spinal cord (Lai et al., 2016), the molecular mechanisms that regulate their distribution remain unknown.

Recent observations in our laboratory indicate that transcription factors of the Onecut (OC) family are present in numerous neuronal populations, including motor neurons and interneurons (Francius and Clotman, 2010; Roy et al., 2012; Francius et al., 2013) and regulate neuronal diversification and migration in the developing spinal cord (Roy et al., 2012; Francius and Clotman, 2014); unpublished data). Onecut factors, namely Hepatocyte Nuclear Factor-6 (HNF-6, or OC-1), OC-2 and OC-3, are transcriptional activators detected in liver, pancreas and CNS during embryonic development (Lemaigre et al., 1996; Landry et al., 1997; Jacquemin et al., 1999, 2003; Vanhorenbeeck et al., 2002). In the CNS, they regulate the production (Espana and Clotman, 2012a), the diversification (Roy et al., 2012; Francius and Clotman, 2014), the distribution (Espana and Clotman, 2012a,b; Audouard et al., 2013) and the maintenance (Espana and Clotman, 2012a,b; Stam et al., 2012) of specific neuronal populations, as well as the formation of neuromuscular junctions (Audouard et al., 2012). In motor neurons, they are required to maintain levels of *Isl1* expression necessary to support the diversification of this population into distinct subsets (Roy et al., 2012). Their presence was also detected in the dorsal embryonic spinal cord (Francius and Clotman, 2010; Francius et al., 2013). However, their distribution in dINs and their possible functions in the development of these cell populations remain unknown. Here, we demonstrate that OC factors are produced in a temporally- and spatially-defined manner in most early populations of spinal dINs. Furthermore, our analyses of mutant mice devoid of OC factors suggest that these proteins control genetic programs that contribute to the regulation of dIN diversification and distribution during embryonic development.

## Materials and Methods

### Animals

All experiments were strictly performed in accordance with the European Community Council directive of 24 November 1986 (86-609/ECC) and the decree of 20 October 1987 (87-848/EEC). Mice were raised in our animal facilities and treated according to the principles of laboratory animal care, and experiments and mouse housing were approved by the Animal Welfare Committee of Université catholique de Louvain (Permit Number: 2013/UCL/MD/11). CD1 or mutant strain mice were crossed and the day of vaginal plug was considered to be embryonic day (e) 0.5. The embryos were collected at e10.5, e11.5 and e12.5, a minimum of three embryos of the same genotype were used in each experiment. *Hnf6* and *Oc2* mutant mice have been described previously (Jacquemin et al., 2000; Clotman et al., 2005).

### Immunofluorescence Labelings

Collected embryos were fixed in ice-cold 4% paraformaldehyde (PFA) in phosphate buffered-saline (PBS) for 10–30 min according to their embryonic stage, incubated in PBS/30% sucrose solution overnight at 4°C, embedded and frozen in PBS/15% sucrose/7.5% gelatine. Immunostaining was performed on 12 μm serial cryosections as previously described (Francius and Clotman, 2010). Primary antibodies against the following proteins were used: Arx (rabbit; 1:1000; kindly provided by J. Chelly), Bhlhb5 (goat; 1:700; Santa Cruz #sc-6045), Brn3 (goat; 1:500; Santa Cruz #sc-6026), Brn3a (guinea pig; 1:2000; kindly provided by J.E. Johnson; or mouse 1:1000; Santa Cruz #sc-8429), Dmrt3 (guinea pig; 1:5000; kindly provided by K. Kullander #170), Foxd3 (guinea pig; 1:5000; or rabbit; 1:2000; kindly provided by T. Müller), Foxp1 (goat; 1:1000; R&D Systems #AF4534), Foxp2 (goat; 1:1000; Abcam #ab1307), β-galactosidase (chicken; 1:2000; Abcam #ab9361), HNF6 (guinea pig; 1:2000; (Espana and Clotman, 2012b); or rabbit; 1:100; Santa Cruz #sc-13050; or sheep; 1:1000 R&D Systems #AF6277), Isl1/2 (goat; 1:3000; Neuromics #GT15051; or mouse; 1:6000; DSHB #39.4D5), Lbx1 (guinea pig; 1:10,000; or rabbit; 1:5000; kindly provided by T. Müller), Lhx1/5 (mouse; 1:1000; DSHB #4F2), Lmx1b (guinea pig; 1:10,000; or rabbit; 1:2000; kindly provided by T. Müller; or armenian hamster; 1:2; kindly provided by Y. Ono), MafB (rabbit; 1:5000; kindly provided by H. Wende), cMaf (rabbit; 1:3000; kindly provided by H. Wende), OC2 (rat; 1:400; (Clotman et al., 2005); or sheep; 1:500; R&D Systems #AF6294), OC3 (guinea pig; 1:6000; (Pierreux et al., 2004)), mOct6/Scip/Pou3f1 (rabbit; 1:50; kindly provided by Dies N. Meijer), Nurr1 (rat; 1:2000; kindly provided by Y. Ono), Olig3 (guinea pig; 1:6000; or rabbit; 1:2000; kindly provided by T. Müller), Otp (rabbit; 1:500; kindly provided by F. Vaccarino), Pax2 (rabbit; 1:1000; Covance PRB-276P), Phox2a (rabbit; 1:500; kindly provided by J.-F. Brunet), Prox1 (rabbit; 1:1000; Covance PRB-238C), Tlx3 (guinea pig; 1:10,000; or rabbit; 1:2000; kindly provided by T. Müller), Wt1 (rabbit; 1:2000; Santa Cruz #sc-192). Secondary antibodies donkey anti-guinea pig/AlexaFluor 488, 594 or 647, anti-mouse/AlexaFluor 488, 594 or 647, anti-rabbit/AlexaFluor 594 or 647, anti-goat/AlexaFluor 488, anti-rat/AlexaFluor 488 or 647, anti-sheep/AlexaFluor 594, anti-armenian hamster/AlexaFluor 594, purchased from ThermoFisher Scientific or Jackson Laboratories were used at dilution 1:2000.

### Cell Quantification

Immunofluorescence images of cryosections were acquired on Zeiss Axio Cell Observer Z1 confocal microscope, EVOS FL or EVOS FL Auto Cell Imaging System. Brightness and contrast were adjusted uniformly in all replicate panels within an experiment with Adobe Photoshop CS3 software to match with observations. Cell quantification was performed using Adobe Photoshop CS3 software count analysis tool. Labeled neurons were counted on both sides of the spinal cord, on three sections for Onecut quantifications and on five sections for control/mutant comparisons, in brachial or thoracic regions from at least three pairs of control/mutant embryos. Raw data were exported from Adobe Photoshop CS3 software to SigmaPlot v11.0 software and processed to generate histogram figures and statistical analyses.

### Spatial Distribution

Pictures for quantification of interneuron spatial distribution were acquired on an EVOS FL Auto Cell Imaging System or a LEICA TCS confocal microscope. Distance and angle were measured using ruler analysis tool in Adobe Photoshop CS5 software. Spinal cord height (*H*) was defined as the distance from the ventral limit of central canal to the dorsal-most point of spinal cord, and width (*W*) as the distance from central canal to the most lateral edge (adapted from Palmesino et al., 2010). For each dIN, distance (*D*_IN_) and angle (*α*_IN_) were measured from the ventral limit of the central canal to the interneuron soma. DV and medio-lateral (ML) position of dINs were expressed as percentage of spinal cord height and hemicord width respectively: DV position and ML position were defined as (*D*_IN_ * sin*α*_IN_)/*H* and (*D*_IN_ * cos*α*_IN_)/*W* (Palmesino et al., 2010). ML vs. DV values were plotted using Matlab software R2013a (Mathworks, Canada). Statistical analyses of dIN distribution were performed using a two-sample Hotelling’s T2, which is a two-dimensional generalization of the Student’s *t* test, as described for similar data sets (Palmesino et al., 2010). The analysis was implemented using the NCSS software package.

## Results

### OC Factors Are Transiently Detected in Spinal Dorsal Interneurons during Development

In the embryonic spinal cord, the OC transcription factors are present in motor neurons and in several populations of ventral interneurons at the onset of neuronal differentiation (Francius and Clotman, 2010; Stam et al., 2012; Francius et al., 2013). These proteins are also detected in more dorsal populations located in the mantle zone (Francius and Clotman, 2010), suggesting that they are present in differentiating dINs. To address this question, we analyzed the distribution of OC factors at thoracic levels in the dorsal embryonic spinal cord at early stages of dIN differentiation, namely e10.5 and e12.5. Dorsal populations were identified by immunofluorescence using combinations of specific markers, and the presence of HNF-6, OC-2 and OC-3 in each population was individually addressed and quantified. Immunolabelings are illustrated at thoracic levels of the spinal cord, similar observations were made at brachial and lumbar levels.

**Figure 1:**
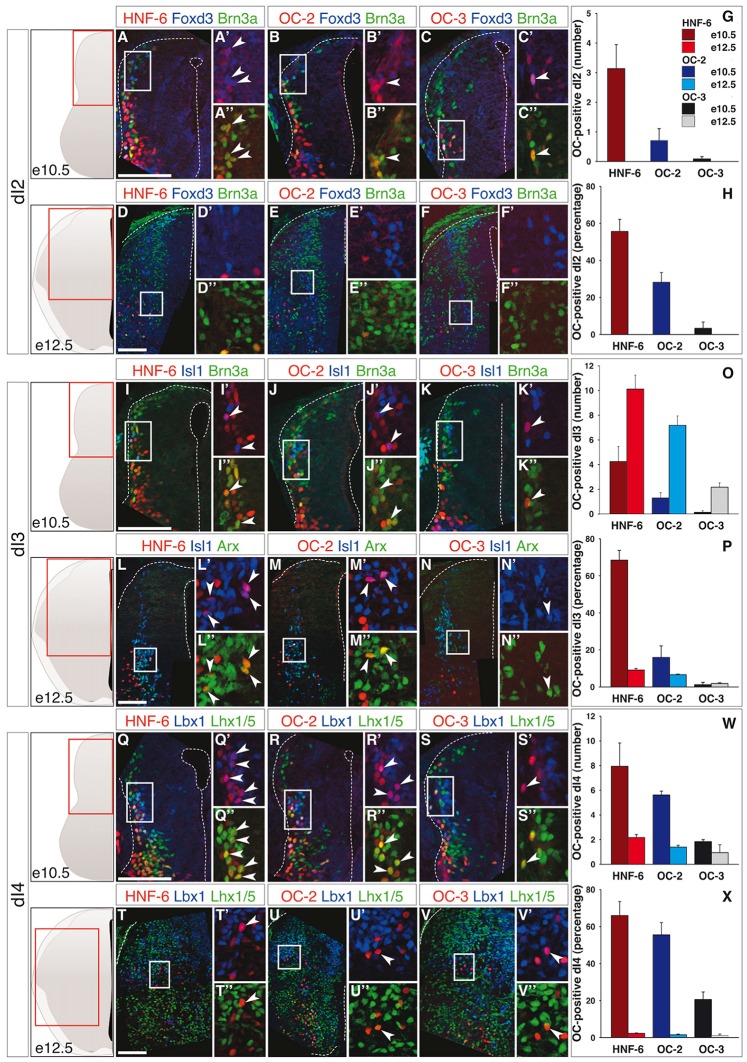
**Onecut (OC) factors are differentially and dynamically distributed in dI2 to dI4 populations. (A–F)** Immunodetection of HNF-6, OC-2 or OC-3 (red) in Foxd3^+^ (blue) Brn3a^+^ (green) dI2 neurons on transverse sections of thoracic spinal cord at e10.5 **(A–C)** and e12.5 **(D–F)**. HNF-6 **(A–A”)**, OC-2 **(B–B”)** and OC-3 **(C–C”)** are visible in a few dI2 cells at e10.5 whereas they are not detected at e12.5 **(D–F”)**. The number **(G)** and percentage **(H)** of OC-positive dI2 neurons per hemisection were quantified accordingly. **(I–N)** Immunodetection of HNF-6, OC-2 or OC-3 (red) in Isl1^+^ (blue) Brn3a^+^/Arx^+^ (green) dI3 neurons on transverse sections of thoracic spinal cord at e10.5 **(I–K)** and e12.5 **(L–N)** demonstrates that OC factors are present in dI3 from e10.5 to e12.5 with a slight predominance of HNF-6 **(I,L)** compared to OC-2 **(J,M)** or OC-3 **(K,N)**. Despite an increase in the number of OC^+^ dI3 neurons from e10.5 to e12.5 **(O)**, the percentage of dI3 neurons containing OC factors **(P)** is reduced at embryonic stage e12.5. **(Q–V)** Immunodetection of HNF-6, OC-2 or OC-3 (red) in Lbx1^+^ (blue) Lhx1/5^+^ (green) dI4 neurons on transverse sections of thoracic spinal cord at e10.5 **(Q–S)** and e12.5 **(T–V)** shows that OC factors are present in early dI4 neurons, HNF-6 **(Q–Q”)** being the most represented. At e12.5, HNF-6 **(T–T”)** and OC-2 **(U–U”)** are still detected in a few dI4 neurons while OC3 **(V–V”)** is barely detectable. Quantifications at embryonic stages e10.5 and e12.5 show that number **(W)** and percentage **(X)** of OC-positive dI4 neurons decrease from e10.5 to e12.5. The pictures show left hemisections as indicated to the left. Dashed lines delineate the spinal cord and the lumen of the ventricle. Insets are magnified views of boxed regions. Arrowheads point to double-labeled cells. Mean values ±SEM, *n* ≥ 3. Scale bars = 100 μm.

As previously reported in motor neurons and ventral interneurons (Francius and Clotman, 2010; Stam et al., 2012; Francius et al., 2013), the distribution of the three Onecut proteins in dINs was partially overlapping. Accordingly, subsets of cells in each dorsal population except dI1 interneurons (data not shown) contained either one, two or the three OC factors (Supplementary Figures S1, S2A,C). Newly-born dI2 cells express *Lhx1* and *Olig3* (Helms and Johnson, 2003; Müller et al., 2005), and HNF-6 was detected as early as e9–9.5 in a majority of these cells (Supplementary Figure S3). At later stages, dI2 interneurons are identified as Foxd3- and Brn3a-positive cells (Helms and Johnson, 2003) in dorsal and medial spinal cord. At e10.5, the three OC factors were detected in a limited number of dI2 cells (Figures 1A–C,G), although corresponding to a significant fraction of the differentiating dI2 interneurons (Figure 1H). However, the prevalence of dI2 containing OC factors quickly decreased and OC were completely absent from dI2 cells at e12.5 (Figures 1D–H).

The dI3 interneurons are defined by the presence of Isl1, Brn3a and Arx (Helms and Johnson, 2003; Poirier et al., 2004). The number of dI3 cells containing OC factors increased from e10.5 to e12.5 (Figures 1I–O), HNF-6 and OC-2 were present in a significant portion of these cells at e10.5 whereas this proportion remained below 10% at e12.5 and at both stages for OC-3 (Figure 1P). As strikingly evidenced at e12.5, most of those dI3 neurons containing OC proteins were located ventrally (Figures 1L–N).

The three OC factors were also present in dI4 interneurons, a dorsal population characterized by combined expression of Lbx1 and Lhx1/5, which is also found in more ventral dI6 cells (Helms and Johnson, 2003). Although OC were detected in a limited number of dI4 (Figures 1Q–V), these corresponded to a significant proportion of these interneurons at e10.5, which sharply decreased at e12.5 (Figure 1W).

**Figure 2:**
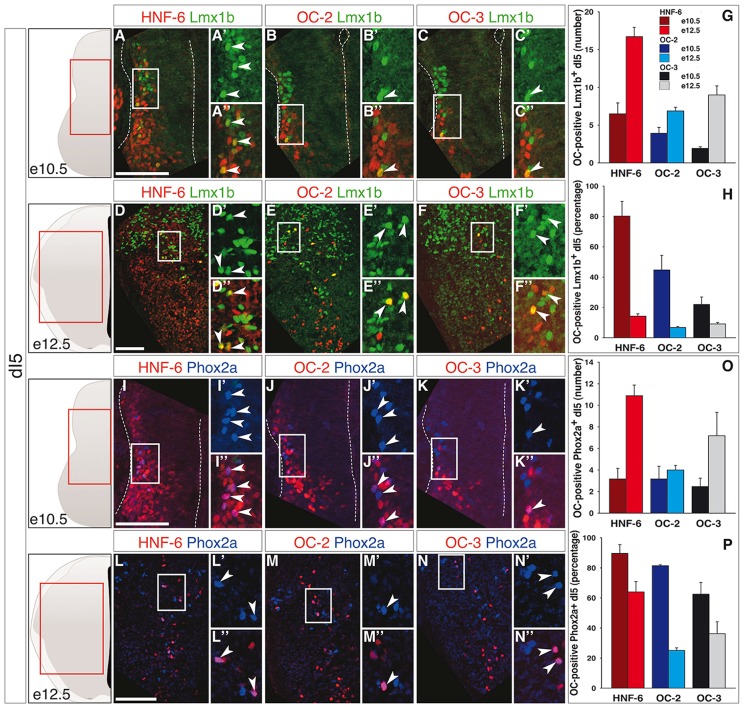
**OC factors are differentially and dynamically distributed in dI5 population and sub-population during development. (A–F)** Immunodetection of HNF-6, OC-2 or OC-3 (red) in Lmx1b^+^ (green) dI5 population on transverse sections of thoracic spinal cord at e10.5 **(A–C)** and e12.5 **(D–F)**. At e10.5, HNF-6 **(A–A”)**, OC-2 **(B–B”)** and OC-3 **(C–C”)** are detected in a large number of dI5 neurons. At e12.5, the presence of HNF-6 **(D–D”)**, OC-2 **(E–E”)** and OC-3 **(F–F”)** is still observed in a few Lmx1b^+^ dI5 neurons. **(G–H)** Quantifications of dI5 neurons containing HNF-6, OC-2 or OC-3 at embryonic stages e10.5 and e12.5 show that HNF-6 is broadly present in early Lmx1b^+^ dI5 neurons at e10.5 while OC-2 and OC-3 are less represented. Between e10.5 and e12.5, the number of dI5 neurons containing OC factors increases **(G)** but the ratio of those OC-positive neurons within Lmx1b^+^ dI5 population decreases **(H)**. **(I–N)** Immunodetection of HNF-6, OC-2 or OC-3 (red) in Phox2a^+^ (blue) dI5 subpopulation on transverse sections of thoracic spinal cord at e10.5 **(I–K)** and e12.5 **(L–N)**. At e10.5, HNF-6 **(I–I”)**, OC-2 **(J–J”)** and OC-3 **(K–K”)** are detected in most of Phox2a^+^ dI5. At e12.5, HNF-6 **(L–L”)**, OC-2 **(M–M”)** and OC-3 **(N–N”)** are still visible in a significant number of Phox2a^+^ interneurons. **(O–P)** Quantifications show an extended presence of OC factors in Phox2a subpopulation at e10.5 **(P)**. As observed in Lmx1b^+^ dI5 population, the number of OC-positive cells increases between stages e10.5 and e12.5 **(O)**, along with a moderate decrease in the ratio of Phox2a^+^ neurons containing OC factors **(P)**. The pictures show left hemisections as indicated to the left. Dashed lines delineate the spinal cord and the lumen of the ventricle. Insets are magnified views of boxed regions. Arrowheads point to double-labeled cells. Mean values ±SEM, *n* ≥ 3; color code as for Figure 1. Scale bars = 100 μm.

The early-born dI5 interneurons and, from e11 onwards, late-born dIL^B^ neurons are identified by Lmx1b (Helms and Johnson, 2003; Ding et al., 2004). At e10.5, OC were present in a restricted number of dI5 interneurons (Figures 2A–C,G) corresponding to a large portion of this population for HNF-6, whereas the distribution of OC-2 and OC-3 was less extended (Figure 2H). As seen in the dI3 population, the number of dI5-containing OC factors rose from e10.5 to e12.5 (Figures 2D–G). However, due to intensive production of dI5 neurons in this time period, the proportion of OC-containing dI5 cells sharply decreased at e12.5 (Figure 2H). These observations suggested that OC factors might be present in a subpopulation of dI5 interneurons generated at early developmental stages. To address this possibility, we investigated the distribution of OC factors in the dI5 subset characterized by the presence of Phox2a (Qian et al., 2002; Ding et al., 2004). At both stages, OC were detected in a number of Phox2a-positive neurons (Figures 2I–O) that corresponded to a majority of these cells at e10.5 and to a lower but significant proportion at e12.5 (Figure 2P). Thus, OC factors are present in dI5 interneurons and preferentially allocated to the Phox2a-positive dI5 subset generated at early developmental stages.

**Figure 3:**
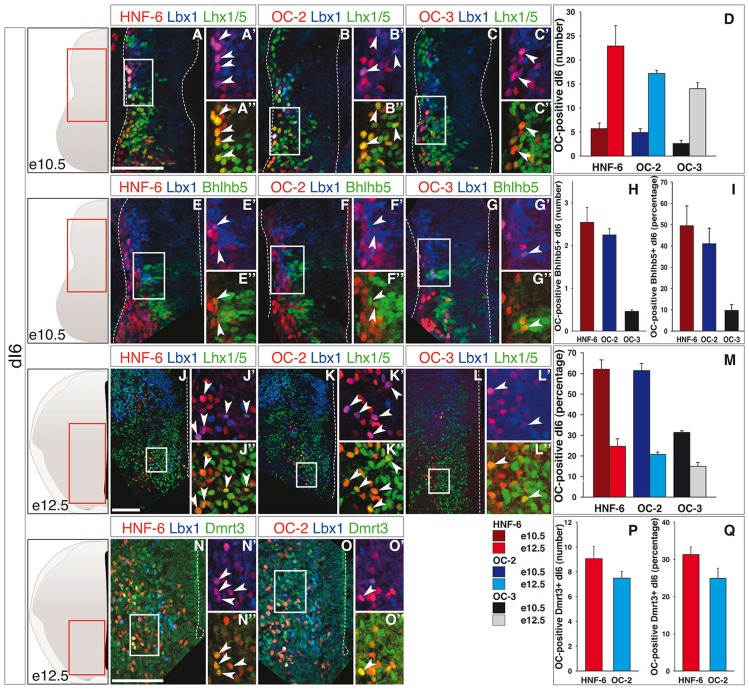
**OC factors are differentially and dynamically distributed in dI6 population and sub-populations during development. (A–I)** Immunodetection of HNF-6, OC-2 or OC-3 (red) in double-labeled Lbx1^+^ (blue)-Lhx1/5^+^ (green, **A–C**) dI6 population or in Bhlhb5^+^ (green, **E–G**) dI6 subpopulation on transverse sections of thoracic spinal cord at e10.5. HNF-6 **(A,E)** and OC-2 **(B,F)** are present in a majority of Lbx1^+^Lhx1/5^+^ dI6 neurons and in a significant number of Bhlhb5^+^ newly-born dI6 while OC-3 **(C–G)** is limited to a smaller number of cells. The number **(D–H)** and percentage **(I–M)** of OC-positive dI6 neurons per hemisection were quantified accordingly. **(J–Q)** Immunodetection of HNF-6, OC-2 or OC-3 (red) in double-labeled Lbx1^+^ (blue)-Lhx1/5^+^ (green, **J–L**) dI6 neurons or in Dmrt3^+^ (green, **N,O**) dI6 subpopulation on transverse sections of thoracic spinal cord at e12.5. HNF-6 **(J,N)** and OC-2 **(K,O)** are mainly detected in dI6 neurons located in ventral spinal cord. OC-3 **(L)** is also detected in Lbx1^+^Lhx1/5^+^ dI6 neurons. Its presence in Dmrt3^+^ dI6 could not be evaluated due to incompatibility between antibody species. **(D,M)** Quantifications of ventrally located dI6 neurons containing HNF-6, OC-2 or OC-3 at e12.5 show an important increase in the number of dI6 containing OC factors from stage e10.5 to e12.5 **(D)** but the percentage of dI6 containing OC decreases **(M)**. **(P,Q)** The number of Dmrt3^+^ dI6 containing HNF-6 and OC-2 is small. The pictures show left hemisections as indicated to the left. Dashed lines delineate the spinal cord and the lumen of the ventricle. Insets are magnified views of boxed regions. Arrowheads point to double-labeled cells. Mean values ±SEM, *n* ≥ 3. Scale bars = 100 μm.

Finally, the dI6 population is defined by combined expression of Lbx1 and Lhx1/5 (Helms and Johnson, 2003) and includes Bhlhb5-expressing early-born dI6 subpopulation at e10.5 (Liu et al., 2007) and Dmrt3- and WT1-positive subsets at e12.5 (Goulding, 2009; Vallstedt and Kullander, 2013). At e10.5, OC factors were detected in a limited number of dI6 interneurons (Figures 3A–D), although corresponding to a significant portion of this population (Figure 3M). As observed for dI3 and dI5, the amount of OC-positive dI6 increased from e10.5 to e12.5 (Figures 3J–L,D) but the proportion of these cells in the global dI6 population significantly decreased (Figure 3M). In addition, OC were present in part of the Bhlhb5-positive dI6 neurons at e10.5 (Figures 3E–I) and in a fraction of the Dmrt3-positive subset at e12.5 (Figures 3N–Q), without any significant enrichment in any of these two subpopulations (compare Figure 3M with Figures 3I,Q, respectively). OC factors were never detected in WT1-positive dI6 interneurons (Supplementary Figure S4).

Hence, OC factors were transiently detected in subsets of early-born dIN populations from dI2 to dI6 during the early steps of differentiation. Their distribution was generally broader and more persistent in more ventral dI populations as compared to dorsal cells, suggesting a possible repression of their expression by DV signaling gradients that contribute to dIN development. As previously observed in other neuronal populations (Francius and Clotman, 2010; Chakrabarty et al., 2012; Espana and Clotman, 2012a,b; Roy et al., 2012; Stam et al., 2012), the three OC proteins displayed a partially overlapping distribution (Figures 1–3; Supplementary Figures S1, S2A,C). In addition, the hierarchy in their spatial extent (HNF-6 > OC-2 > OC-3) previously reported in other populations (Francius and Clotman, 2010; Espana and Clotman, 2012b) was also conserved, except for dI5 at e12.5 wherein OC-3 was more extended than OC-2. Thus, OC factors are dynamically and differentially distributed in early spinal dIN populations, suggesting that they may participate in some aspects of dIN development.

### Onecut Factors Regulate the Distribution of dI3 Interneurons

To assess whether the OC proteins contribute to the development of dINs, we analyzed their phenotype in mouse embryos lacking OC factors, namely *Hnf6/Oc2* double-mutant embryos. Indeed OC-3 is undetectable in the spinal cord of these mutants (Supplementary Figure S2; Roy et al., 2012), suggesting that HNF-6 and OC-2 cooperatively regulate *Oc3* expression in the developing spinal cord. Considering possible differences at specific levels along the antero-posterior axis (Francius et al., 2013), analyses were performed at anterior limb (brachial region) and at thoracic levels. Since OC factors are barely detectable in the most dorsal dI1 and dI2 populations (Figures 1A–H and Supplementary Figure S3), we first addressed their potential role in dI3 development. Interestingly, dI3 is the single dIN population characterized by the presence of the LIM homeodomain protein Isl1, and OC factors are required in motor neurons to maintain proper *Isl1* expression levels throughout differentiation (Roy et al., 2012).

**Figure 4:**
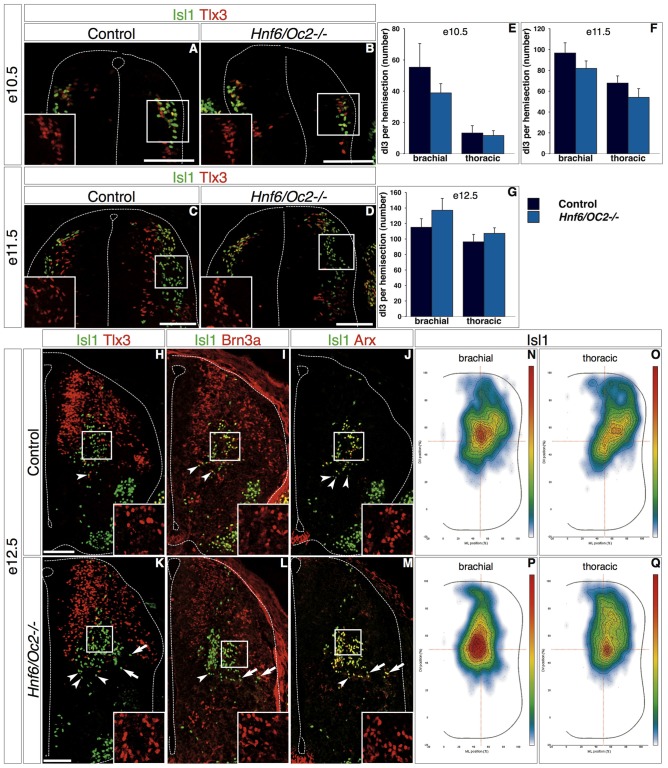
**OC factors control dI3 distribution. (A–M)** Differentiation and distribution of dI3 interneurons in control or *Hnf6/Oc2-/-* embryos. At e10.5 **(A,B)** and e11.5 **(C,D)**, dI3 interneurons containing Isl1 and Tlx3 in *Hnf6/Oc2-/-* embryos **(B,D)** are detected in numbers and distributions comparable to those observed in control embryos **(A,C)**. Tlx3+Isl1- cells next to the ventricular zone in e11.5 control embryo are late-born dILB **(C)**. **(E–F)** Quantitative analysis of control or *Hnf6/Oc2-/-* embryos at e10.5 (**E**, *n* = 4) and e11.5 (**F**, *n* = 4) shows no statistically significant difference in the number of dI3 cells. At e12.5, Isl1 combined with Tlx3, Brn3a or Arx identifies dI3 interneurons in the central intermediate spinal cord of control **(H–J)** or *Hnf6/Oc2-/-* **(K–M)** embryos. The number of dI3 cells is comparable in control and mutant embryos, and Tlx3, Brn3a and Arx are detected in the Isl1-positive cells (insets in **H–M**) indicating that the differentiation of dI3 interneurons is not affected by the absence of Onecut factors. Quantitative analysis shows similar numbers of dI3 cells in control or mutants embryos (**G**, control *n* = 6, mutant *n* = 7). However, the distribution of these cells seems different in *Hnf6/Oc2-/-* embryos (arrows). **(N–Q)** Density plots of dI3 interneuron distribution at brachial **(N,P)** or thoracic **(O,Q)** levels in control **(N,O)** or *Hnf6/Oc2-/-* mutant **(P,Q)** spinal cord at e12.5 show an alteration in the localization of dI3 cells in mutant embryos. The dI3 interneurons migrate as a more compact population in a general dorso-medial to ventro-medial direction, with the densely-packed cells closer to the migration front. Analysis was performed on at least three sections from five control and six mutant embryos (*n* > 2600 cells per condition; *p* ≤ 0.001). The pictures show left hemisections. Dashed lines delineate the spinal cord and the lumen of the ventricle. Insets are magnified views of boxed regions and show Tlx3, Brn3a and Arx labeling, respectively. Arrowheads point to properly-located dI3 neurons. Arrows indicate unproperly-located dI3 neurons in the *Hnf6/Oc2-/-* mutant embryos. Mean values ±SEM. Scale bars = 100 μm.

At e10.5 in control embryos, dI3 interneurons co-labeled for Isl1 and Tlx3 were located in a dorsal portion of the developing spinal cord, part of the population in register with the pdI3 progenitor domain while some cells did initiate ventral migration and extended to the Tlx3-single labeled dI5 domain (Figure 4A). In *Hnf6/Oc2-/-* mutant embryos, Tlx3 and Isl1 were co-detected in a dorsal population similarly distributed from the pdI3 DV position to the location of dI5 interneurons (Figure 4B), indicating that dI3 interneurons are produced in *Oc* mutant embryos and that OC factors are not required for *Isl1* expression in these cells. The amount of dI3 cells was similar to that observed in control littermates, as confirmed by cell quantifications (Figure 4E). At e11.5, the number of control dI3 interneurons had increased and cells were distributed as a lateral stream extending from the pdI3 position to intermediate dorsoventral levels of the spinal cord (Figure 4C). In *Hnf6/Oc2-/-* embryos, dI3 interneurons similarly extended from dorsal regions to the middle of the spinal cord and their number was preserved (Figures 4D, 4F). At e12.5, to ascertain the identity of the cells that migrated in the ventral regions, dI3 interneurons were additionally labeled for Brn3a, which is detected in dI1/2/3/5/L^B^, and for Arx present in dI3 cells and V1 ventral interneurons (Francius et al., 2013). In control embryos, co-detection of these markers identified the dI3 population in the central intermediate region of the spinal cord, with a few dI3 cells migrating towards the floor plate (Figures 4H–J, insets show Tlx3, Brn3a and Arx labeling, respectively), consistent with their final location in the deep dorsal horn and in the intermediate laminae (Ding et al., 2005; Müller et al., 2005; Bui et al., 2015). In *Hnf6/Oc2-/-* embryos, dI3 interneurons were detected in similar regions of the spinal cord and the number of cells was comparable to that observed in controls (Figures 4K–M), as confirmed by quantifications (Figure 4G). Noticeably, the presence of the dI3 markers including Isl1 was not affected by the absence of OC proteins (Figures 4K–M, insets show Tlx3, Brn3a and Arx labeling, respectively), indicating that Isl1 maintenance in dI3 cells and proper differentiation of dI3 interneurons do not require OC factors. However, the distribution of these cells seemed different in the *Hnf6/Oc2-/-* embryos (Figures 4H–M).

Therefore, we decided to investigate the distribution of the whole dI3 population by adapting for interneurons a two-dimensional position analysis initially developed for motor neurons (Palmesino et al., 2010). Using this method, we observed that dI3 interneurons in control embryos migrated as a relatively loose population in an overall dorso-lateral to ventro-medial direction with highest density observed in the center of the distribution area (Figures 4N–O). In contrast, the dI3 interneurons in *Hnf6/Oc2-/-* embryos migrated as a more compact population in a general dorso-medial to ventro-medial direction, with the densely-packed cells close to the migration front (Figures 4P–Q). Taken together, these observations indicate that OC factors are not required for proper differentiation of dI3 interneurons but regulate aspects of their distribution.

### Onecut Factors Regulate Diversification and Distribution of dI5 Subset and Distribution of dI6 Subpopulations

**Figure 5:**
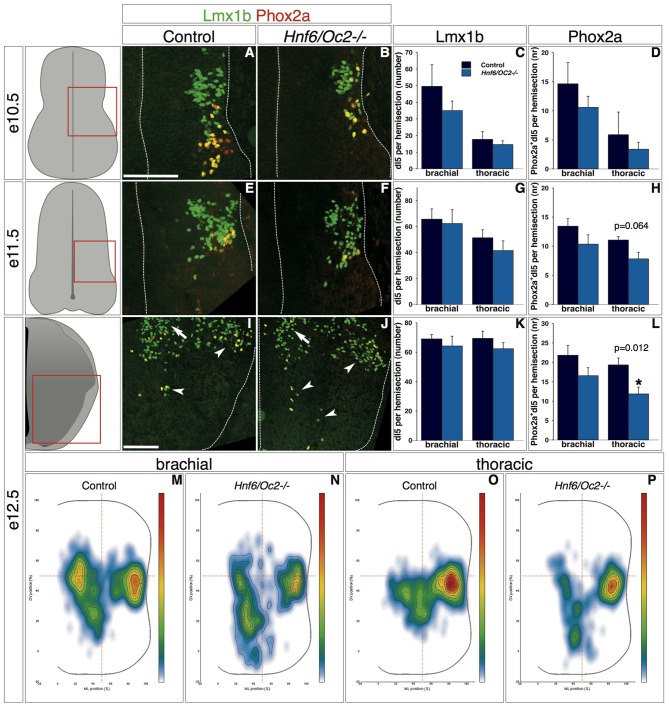
**OC factors control the number and the distribution of Phox2a^+^ dI5 cells. (A–L)** Identification of dI5 interneurons and Phox2a+ dI5 subset in control or *Hnf6/Oc2-/-* embryos. At embryonic stage e10.5 **(A,B)**, Lmx1b is present in dI5 interneurons and Phox2a is detected in a small subset of dI5 cells. **(C,D)** Quantitative analysis shows that number of Lmx1b+ and of Lmx1b+Phox2a+ dI5 neurons at brachial or thoracic levels are comparable in control or *Hnf6/Oc2-/-* mutant embryos (*n* = 4). At e11.5 **(E,F)** and e12.5 **(I,J)**, most Lmx1b+ dI5 are located medio-laterally or migrating ventrally whereas Lmx1b+ late-born dILB extend dorsally along the ventricular zone. Phox2a labeling is still restricted to a medio-lateral (ML) subset of dI5 neurons at e11.5 but laterally and ventrally disperses (arrowheads) at e12.5. **(G,H,K,L)** Quantitative analysis shows similar numbers of Lmx1b+ and Lmx1b+Phox2a+ dI5 cells in control and in mutant embryos at embryonic stage e11.5 (*n* = 3) and e12.5 (*n* = 4 for control and *n* = 7 for mutant embryos) except for a significant decrease of Phox2a+ dI5 subset at thoracic level of e12.5 mutant embryo (*p* = 0.011). **(M–P)** Density plots of Phox2a+ dI5 interneuron distribution at brachial or thoracic levels of control or *Hnf6/Oc2-/-* mutant spinal cord at e12.5. Phox2a+ dI5 cells in the medial cluster are less densely-packed than in control spinal cord but instead spread along the dorso-ventral (DV) axis of the spinal cord. Analysis performed on at least three sections from five control and six mutant embryos (*n* > 250 cells analyzed per condition; *p* ≤ 0.001). The pictures show left hemisections as indicated to the left. Dashed lines delineate the spinal cord and the lumen of the ventricle. Arrowheads point to lateral and ventral groups of dI5 neurons. Arrows show the medial cluster of dI5 cells. Mean values ±SEM. Scale bars = 100 μm.

Due to the lack of specific markers and to perturbations in the distribution of several dI populations in *Hnf6/Oc2-/-* embryos (see above and below), the discrimination between early- and late-born populations of class B dINs was extremely difficult to rigorously achieve in mutant spinal cord. To circumvent this limitation, we restricted our analyses to dI5 and dI6 subsets wherein OC factors were detected (Figures 2, 3). In control embryos at e10.5, Lmx1b-positive interneurons were located in the ML region of the spinal cord, with the Phox2a dI5 subset corresponding to the most ventral cell complement (Figure 5A). In *Hnf6/Oc2-/-* embryos, Lmx1b-positive interneurons and the Phox2a subset were detected in a similar distribution (Figure 5B). Although the number of cells in each subpopulation seemed lower than in control littermates (Figure 5B), this difference was not statistically significant (Figures 5C–D). At e11.5 in control individuals, Lmx1b-positive interneurons devoid of Phox2a were migrating in a medio-ventral direction while the Phox2a subset remained in a ML position (Figure 5E). A similar situation was observed in mutant embryos, without any significant change in cell numbers (Figures 5F–H). At e12.5, Lmx1b-positive cells divided in lateral and medial cell clusters with some medial cells ventrally located. Phox2a-positive cells distributed equally between these different clusters (Figure 5I). In *Hnf6/Oc2-/-* embryos, the number of Lmx1b-positive interneurons and their location were unaffected (Figures 5J–K). However, the number of Phox2a-positive dI5 cells decreased significantly at thoracic level of mutant embryo (Figure 5L; *p* = 0.011) and the relative distribution of these cells in the different clusters seemed different (Figure 5J). This was confirmed by distribution analysis, which showed that Phox2a-positive dI5 interneurons in the medial cluster were less densely-packed but instead spread along the DV axis of the spinal cord (Figures 5M–P).

**Figure 6:**
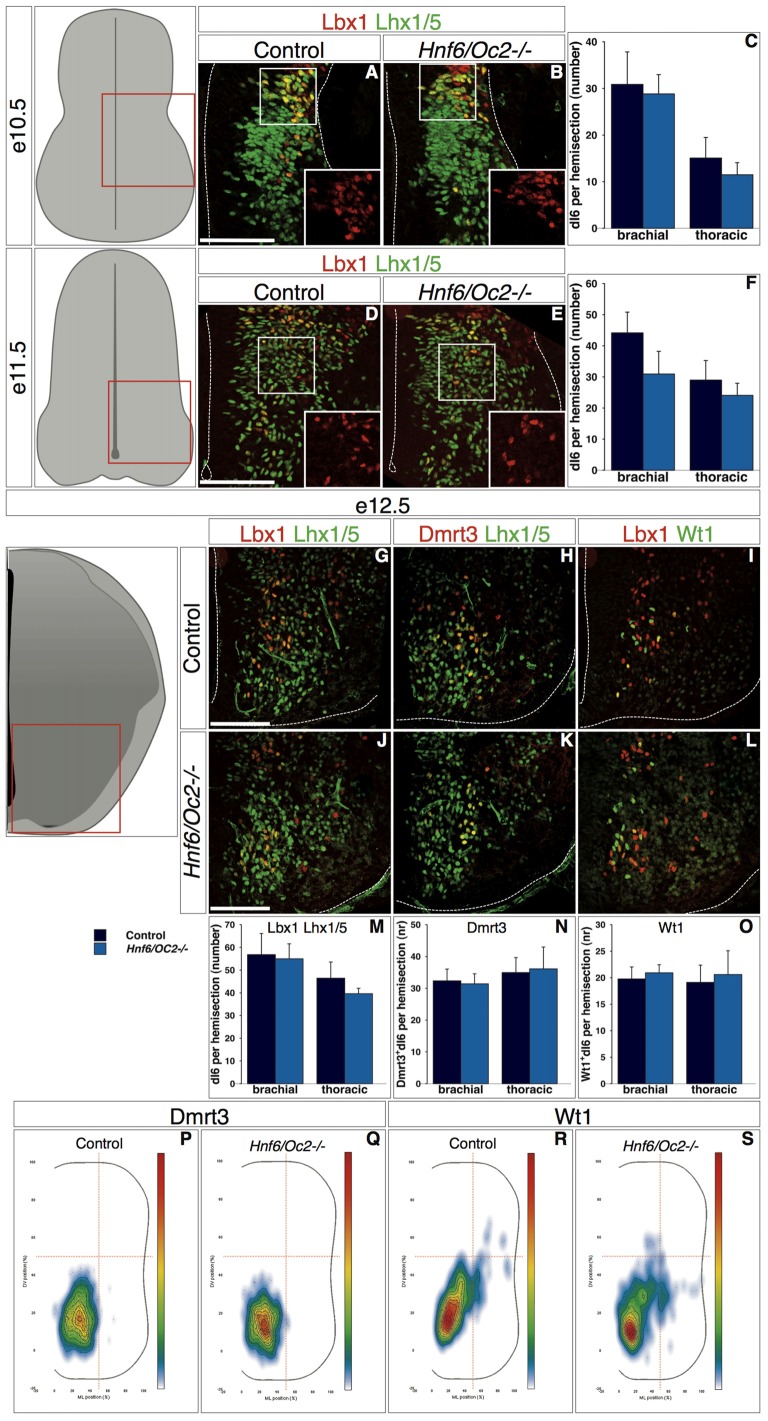
**ContinuedOC factors control dI6 interneuron distribution. (A–O)** Identification of dI6 interneuron population and subpopulations in control or *Hnf6/Oc2-/-* embryos. At the three embryonic stages, dI6 interneurons were identified as ventral Lbx1 + Lhx1/5+ cells. At e10.5, most dI6 interneurons are spread from ventricular zone to lateral deep dorsal spinal cord while fewer are visible in ventral horn (**A,B**; insets show Lbx1 labeling) with comparable amount of cells in control and mutant embryos (**C**, *n* = 4). At e11.5, more dI6 cells are present in lateral and ventral spinal cord of control and Hnf6/Oc2-/- embryos (**D,E**; insets show Lbx1 labeling) without any significant difference in their number (**F**, *n* = 3). At e12.5, Lbx1 + Lhx1/5+ dI6 interneurons are located in ventral regions in control and in mutant embryos **(G,J)**. Two partially overlapping subsets of dI6 population are defined by Dmrt3 **(H,K)** and Wt1 **(I,L)** in the same area. Quantitative analysis of dI6 population or subsets shows comparable numbers of cells at brachial or and at thoracic levels in control or mutant embryos (**M–O**, *n* = 5). **(P–S)** Density plots of Dmrt3+ **(P–Q)** and of Wt1+ **(R–S)** dI6 interneurons at brachial level of control or mutant embryos at stage e12.5. Cells of these dI6 subsets are more densely packed in the ventral region of spinal cord in *Hnf6/Oc2-/-* embryos. Analysis was performed on at least three sections from four (*n* > 760 cells per condition; *p* ≤ 0.001) or five embryos (*n* > 350 cells per condition; *p* ≤ 0.001) for Dmrt3 and Wt1, respectively. The pictures show left hemisections as indicated to the left. Dashed lines delineate the spinal cord and the lumen of the ventricle. Insets are magnified views of boxed regions and show Lbx1 labeling. Mean values ±SEM. Scale bars = 100 μm.

Similarly, early dI6 interneurons in control embryos at e10.5 were initially located in a ML region of the spinal cord (Figure 6A, inset shows Lbx1). Their number and their location were comparable in *Hnf6/Oc2-/-* embryos (Figures 6B,C, inset shows Lbx1). At e11.5 in control embryos, these cells progressively migrated in a medio-ventral direction and intimately intermingled with ventral interneurons characterized by the presence of Lhx1/5 without Lbx1 (Figure 6D, inset shows Lbx1). *Hnf6/Oc2-/-* dI6 interneurons seemed to migrate similarly and their number was not different from control littermates (Figures 6E,F, inset shows Lbx1). At e12.5 in control embryos, dI6 interneurons settled in the ventro-medial region of the spinal cord (Figure 6G). At that stage, two partially overlapping dI6 subsets characterized by the presence of Dmrt3 (Andersson et al., 2012; Vallstedt and Kullander, 2013) or WT1 (Armstrong et al., 1993; Goulding, 2009) could be distinguished. Dmrt3-positive cells gathered in a medio-ventral position (Figure 6H) whereas WT1-positive cells were partly intermingled within and partly extended dorsally to Dmrt3 domain (Figure 6I). The cell number in the dI6 population and in each of these subsets was similar in the absence of OC factors (Figures 6J–O). However, the distribution of each subset was modified. While Dmrt3-positive cells were slightly more densely packed in a more ventral location (Figures 6P–Q), the WT1 complement was also located more ventrally than in control littermates (Figures 6R–S). These observations indicate that the OC factors control some aspects of the diversification of dI5 interneurons and regulate the distribution of dI5 and dI6 subpopulations in the developing spinal cord.

## Discussion

During embryonic development, the dorsal neural tube generates interneuron populations that contribute to motor circuits or that integrate and relay proprioceptive, sensory or nociceptive information to motor circuits or to higher brain centers. The molecular mechanisms regulating the production of those interneurons have been extensively deciphered (Helms and Johnson, 2003; Lewis, 2006; Zhuang and Sockanathan, 2006), but the developmental determinants controlling their diversification, migration and final distribution (Lewis, 2006; Lai et al., 2016) remain elusive. Here, we provide evidence that the OC transcription factors are present in subsets of differentiating dINs and regulate some aspects of the diversification and of the distribution of these populations, likely impacting on neural circuit formation.

In spinal motor neurons and ventral interneurons, OC transcription factors are present in fractions of homogeneous subpopulations irrelevant to their molecular characteristics, neurotransmitter phenotype, location or contribution to neural circuits (Roy et al., 2012; Francius et al., 2013). Similarly, we detected OC factors in multiple dIN subsets including excitatory (dI2, dI3 and dI5) or inhibitory (dI4 and dI6) populations, generated at various levels along DV axis of the neural tube (from dI2 to dI6), involved in modulating motor control (dI3, dI4, dI5 and dI6) or processing sensory information (dI2), and migrating to ventral (dI3, dI5 and dI6) or deep dorsal (dI2 to dI5) locations in the mature spinal cord (Helms and Johnson, 2003; Lewis, 2006; Zhuang and Sockanathan, 2006; Lu et al., 2015; Lai et al., 2016). As for the other neuronal populations wherein OC factors are detected, this raises the question of the upstream regulators generating such a complex and ill-defined expression pattern in the developing CNS. To date, factors controlling *Oc* expression in the spinal cord remain unknown. Furthermore, the cis-acting sequences that stimulate HNF-6 production in the developing liver or pancreas are ineffective in the CNS (Poll et al., 2006), suggesting that *Oc* expression in the neural tissue is regulated by distant enhancers.

Although OC factors are present in differentiating neurons (Francius and Clotman, 2010; Roy et al., 2012; Francius et al., 2013), their distribution in dINs correlates with the DV morphogen gradients known to determine the production of distinct progenitor domains in the dorsal neural tube. Indeed, OC are present in a larger proportion of cells and for a longer period of time in the more ventral populations of dINs than in their dorsal complement. The fraction of dorsal populations containing OC proteins increases from dI2 to dI6 and the number of cells that maintain *Oc* expression at e12.5 also rises from dI2 to dI6 (Figures 1–3). This is reminiscent of the gradient of Wnt and BMP morphogens initially produced by the dorsal ectoderm and subsequently maintained by the roof plate, which determines the identity of the three dorsal-most progenitor compartments without affecting the more ventral populations (Wine-Lee et al., 2004; Timmer et al., 2005; Zechner et al., 2007; Tozer et al., 2013). While expression of receptor Ia to BMP (BMPRIa) is restricted to spinal progenitors, BMPRIb is additionally expressed in the intermediate zone and in differentiating neurons (Yamauchi et al., 2008). Furthermore, Wnt receptors of the Frizzled family are detected at early stages in the whole spinal cord (Hu et al., 2009) and Wnt signaling is active in postmitotic neurons (Galli et al., 2014; Avilés and Stoeckli, 2016). This indicates that, beyond patterning of the neural tube, BMP and Wnt signaling is maintained in differentiating spinal interneurons. Therefore, *Oc* expression may be inhibited in dIN populations by BMP/Wnt signaling that would remain active in differentiating cells. Consistent with this, exposure of human ES cells to Noggin, a BMP antagonist, combined to retinoic acid and FGF-10 increases *Hnf6* expression (Mfopou et al., 2010). In contrast, Wnt1 signaling inhibition by Dkk-1 in ventral midbrain neural stem cells reduces *Hnf6* expression, suggesting that Wnt1 may rather stimulate OC production in neural cells (Yuan et al., 2015). Similar to OC factors, *Pax6* is absent from the most dIN populations because its expression is repressed by BMP signaling (Bel-Vialar et al., 2007). In the retina, OC factors act downstream of Pax6 to maintain horizontal cell identity (Klimova et al., 2015). However, regulation of *Oc* expression by proneural factors or by transcriptional regulators differentially expressed in distinct interneuron populations along the DV axis of the spinal cord has not been reported yet. Hence, identification of the factors upstream of the *Oc* genes in the neural tube constitutes a prerequisite to understand how their complex distribution pattern is achieved in the developing CNS.

Nevertheless, OC regulation must follow stereotyped mechanisms, as similar spatial and temporal progressions are observed among the different neuronal populations wherein OC are detected. Indeed, the distribution area of the three OC proteins follow a HNF-6 > OC-2 > OC-3 hierarchy and *Hnf6* is expressed earlier than *Oc2* detected itself before *Oc3* in spinal motor neurons (Francius and Clotman, 2010; Roy et al., 2012) and ventral interneurons (Francius et al., 2013). A similar distribution dynamic was generally observed in dINs (Figures 1–3), with the single exception for OC-3 detected in a broader fraction of Phox2a-positive dI5 interneurons than OC-2 at e12.5 (Figure 2P). This suggests that similar mechanisms contributing to control *Oc* expression are acting in different cell types, raising the possibility that these factors may regulate generic aspects of neuronal development rather than the acquisition of specific characteristics by distinct neuronal subsets.

However, analysis of the few early dIN subpopulations identified to date suggested that OC factors contribute to dIN diversification. Indeed, the Phox2a subset of dI5 interneurons was reduced in the absence of OC proteins whereas the total number of dI5 cells was maintained. This alteration is not caused by apoptosis, as cell death is not increased in OC mutant embryos (Roy et al., 2012). Lack of markers for other dI5 subsets prevented to investigate whether this resulted from defective production of Phox2a-positive cells or from conversion into other dI5 subtypes. OC factors are known to regulate neuronal diversification in the developing spinal cord. They critically regulate the differentiation ratio between visceral and somatic motor neurons at thoracic levels of the spinal cord and the production of ventral limb innervating motor neurons at brachial and lumbar levels (Roy et al., 2012; Francius and Clotman, 2014). Interestingly, OC control these two developmental events by maintaining significant expression levels of the Lim-homeodomain transcription factors *Isl1* in the differentiating motor neurons (Roy et al., 2012). Isl1 maintenance results from direct stimulation of *Isl1* expression by the OC factors, as evidenced by direct binding of OC proteins (Roy et al., 2012; Kim et al., 2015) to the CREST-2 enhancer of *Isl1* (Uemura et al., 2005) and by the dependence of CREST-2 activity on the OC factors (Roy et al., 2012; Kim et al., 2015). Isl1 is also produced in dI3 interneurons during their differentiation (Liem et al., 1997; Bui et al., 2013), and determines their axonal projections at later developmental stages (Avraham et al., 2010). The CREST-2 enhancer of *Isl1* is active in the dI3 interneurons (Kim et al., 2015), suggesting that OC factors may also regulate *Isl1* expression in dI3 neurons and possibly later steps of dI3 differentiation. However, Isl1 was detected in dI3 neurons in the absence of OC factors and the number of dI3 cells in *Hnf6/Oc2-/-* mutant embryos was comparable to that observed in control littermates, indicating that OC are dispensable for dI3 production and for *Isl1* expression in dI3. This demonstrates that *Isl1* regulation by the OC factors is highly cell-type dependent and suggests that other factors that remain to be identified activate the CREST-2 enhancer in dI3 interneurons independently from OC proteins.

Similarly, OC factors regulate the diversification of spinal ventral interneurons, the production of some ventral interneuron subsets being impaired in the absence of OC proteins (AH, VR and FC, unpublished observations). The characterization of distinct ventral neuronal subsets has been frenetically pursued in the recent years and led to the identification of an extensive, although yet incomplete, collection of dozens of discrete subpopulations with specific molecular, electrophysiological, distribution or functional characteristics (Gosgnach et al., 2006; Zagoraiou et al., 2009; Panayi et al., 2010; Borowska et al., 2013; Dougherty et al., 2013; Francius et al., 2013; Panayiotou et al., 2013; Talpalar et al., 2013; Bikoff et al., 2016). This knowledge provided molecular and genetic tools to specifically identify, record or functionally challenge these different subsets and to characterize their phenotype in mutant embryos. In contrast, only few early dIN subsets have been identified to date, based on molecular markers (Ding et al., 2004; Rebelo et al., 2007; Andersson et al., 2012; Dougherty et al., 2013; Hilde et al., 2016). Thus, a possible involvement of OC proteins in dorsal diversification awaits a more complete characterization of the different subpopulations produced by the known cardinal populations of dINs.

Moreover, our data indicate that the OC factors regulate the distribution of different dIN populations during spinal cord development. Although limited to short-distance cell movements, spinal neuronal migration is a very complex process. On the one hand, each population or subset of motor neurons and of interneurons migrates according to a specific trajectory, in a specific direction, and eventually settles at a specific location. In addition, some populations assume their general migratory trajectory along the ventricular zone as soon as they exit their progenitor domain whereas others migrate first in a radial orientation before turning in ventral or dorsal direction. These combined processes result in an intense intermingling of cells with distinct identities. On the other hand, cells from a single subset do not migrate as a cohesive compact cluster but rather form a relatively loose population that progresses in a common general direction. Eventually, these cells settle at a more or less focused final location depending on each subset. To analyze this highly dynamic and complex process, we adapted a distribution analysis routine initially developed for motor neurons (Palmesino et al., 2010). This routine enabled to obtain a highly reproducible integrated view of a cell population distribution on the transversal plane of the spinal cord and to precisely compare the migration pathway and the final location adopted by molecularly-defined cell populations in wild type or mutant embryos.

Consistent with the presence of OC proteins in small proportions of each dI population, only fractions of dIN subsets were mislocated in *Oc* mutant embryos. Lack of OC does not affect the capacity of cells to migrate nor their intrinsic motility. Indeed, we did not observe any delay in dIN migration nor any premature arrest of migration at inappropriate settling location in *Oc* mutant embryos, suggesting that their migration abilities are conserved. In contrast, three types of migration defects were observed in *Hnf6/Oc2-/-* embryos. First, the migration trajectory of the dI3 interneurons was shifted to a more medial pathway than in control embryos (Figures 4P–Q). Second, the organization of migrating dI3 (Figures 4P–Q) and WT1-positive dI6 interneurons (Figure 6S) was altered, since cells migrated as a more compact cluster with the highest cell density closer to the migration front. Third, Phox2a-positive dI5 interneurons settling position was modified (Figures 5M–P), with a reduced number of cells in the lateral cluster and significant migration in ventro-medial locations where Phox2a-positive dI5 are never found in control embryos. Slight changes in settling location were also observed for dI3 (Figures 4P–Q) and dI6 (Figure 6S) subsets. These observations suggest that the production of environmental cues that determine the migration pathway and the settling location of dIN populations or their interpretation by migrating cells is altered in the absence of OC factors. They also raise the possibility that factors involved in clustering or in cohesion of these migrating populations may be affected, as previously proposed in the dopaminergic A13 nucleus of *Hnf6/Oc2-/-* embryos (Espana and Clotman, 2012b), although changes in cell clustering may be secondary to alterations in the migration of individual cells. The molecular mechanisms regulated by OC actors in these processes remain to be identified.

Requirement of proper neuronal migration for adequate formation of neural circuits in the developing spinal cord has been repeatedly questioned (Kania, 2014). Indeed, some mutant lines show mispositioned neuron cell bodies but proper connectivity of the affected cells and organization of the corresponding neural circuits (Coonan et al., 2003; Demireva et al., 2011). However, increasing amount of evidence demonstrate that the location of spinal neurons is critical for proper neuronal connectivity and adequate integration into sensory-motor circuitries (Sürmeli et al., 2011; Bikoff et al., 2016). In dorsal spinal cord, the spatial organization of Lbx1-derived premotor interneurons determines their role, i.e., flexor interneurons born at e10.5 migrate laterally in lamina VI while extensor interneurons born at e12.5 remain closer to the central canal (Tripodi et al., 2011). EphA4 in these cells determines their localization as well as their axonal projections and their contribution to left-right motor alternation (Satoh et al., 2016). Likewise, the sensory cues that trigger motor responses are linked to the ML position of dorsal premotor interneurons in the deep dorsal horn (Hilde et al., 2016). Therefore, identification of the molecular regulators of neuronal migration and distribution in the spinal cord is important to understand circuit formation and neural activity. Interestingly, the interneuron populations altered in absence of OC factors are involved in motor control, either modulating motor neurons (Andersson et al., 2012; Bui et al., 2013; Goetz et al., 2015; Satoh et al., 2016) or ventral premotor interneuron activity (Levine et al., 2014; Hilde et al., 2016) or mediating presynaptic inhibitory control of proprioceptive sensory neurons (Betley et al., 2009; Fink et al., 2014). Hence, OC factors could have been recruited in different neuronal subsets to coordinate the development of distinct populations eventually involved in motor circuits.

## Author Contributions

KUK, GM, AH and FC designed the experiments. BJ generated Matlab routines for the distribution analyses of dorsal interneurons. KUK, GM, AH and VR performed the experiments. GM performed additional experiments for the revision of the manuscript, and all the authors discussed the data. KUK and FC drafted the manuscript.

## Conflict of Interest Statement

The authors declare that the research was conducted in the absence of any commercial or financial relationships that could be construed as a potential conflict of interest.
