# Supplementary figures and images for: The Onecut Transcription Factors Regulate Differentiation and Distribution of Dorsal Interneurons during Spinal Cord Development
Source: Front Mol Neurosci. 2017 May 26;10:157. doi: 10.3389/fnmol.2017.00157 (PMC5445119; doi:10.3389/fnmol.2017.00157)

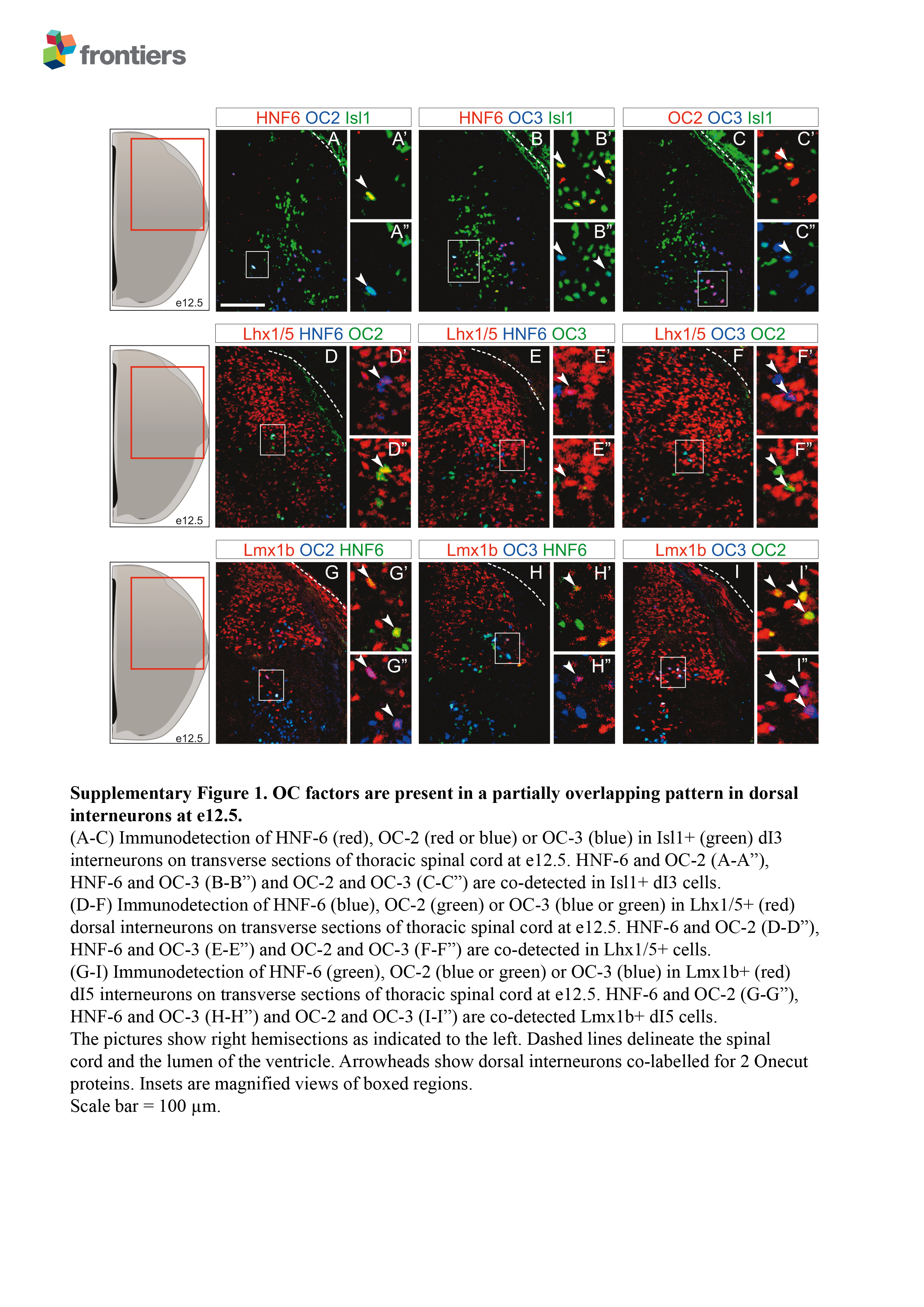

Supplement: Supplementary file 1 [file Image_1.jpeg]

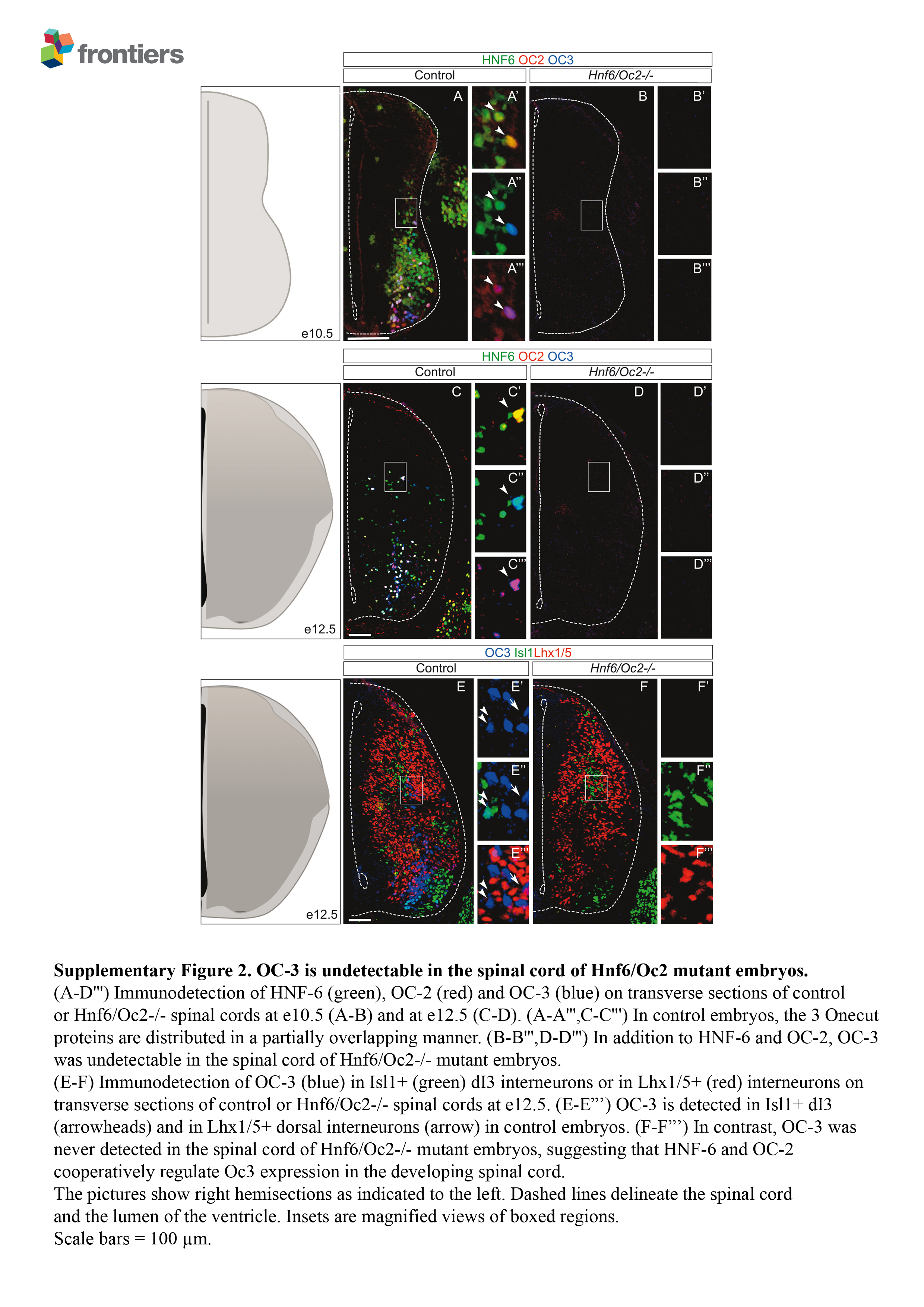

Supplement: Supplementary file 2 [file Image_2.jpeg]

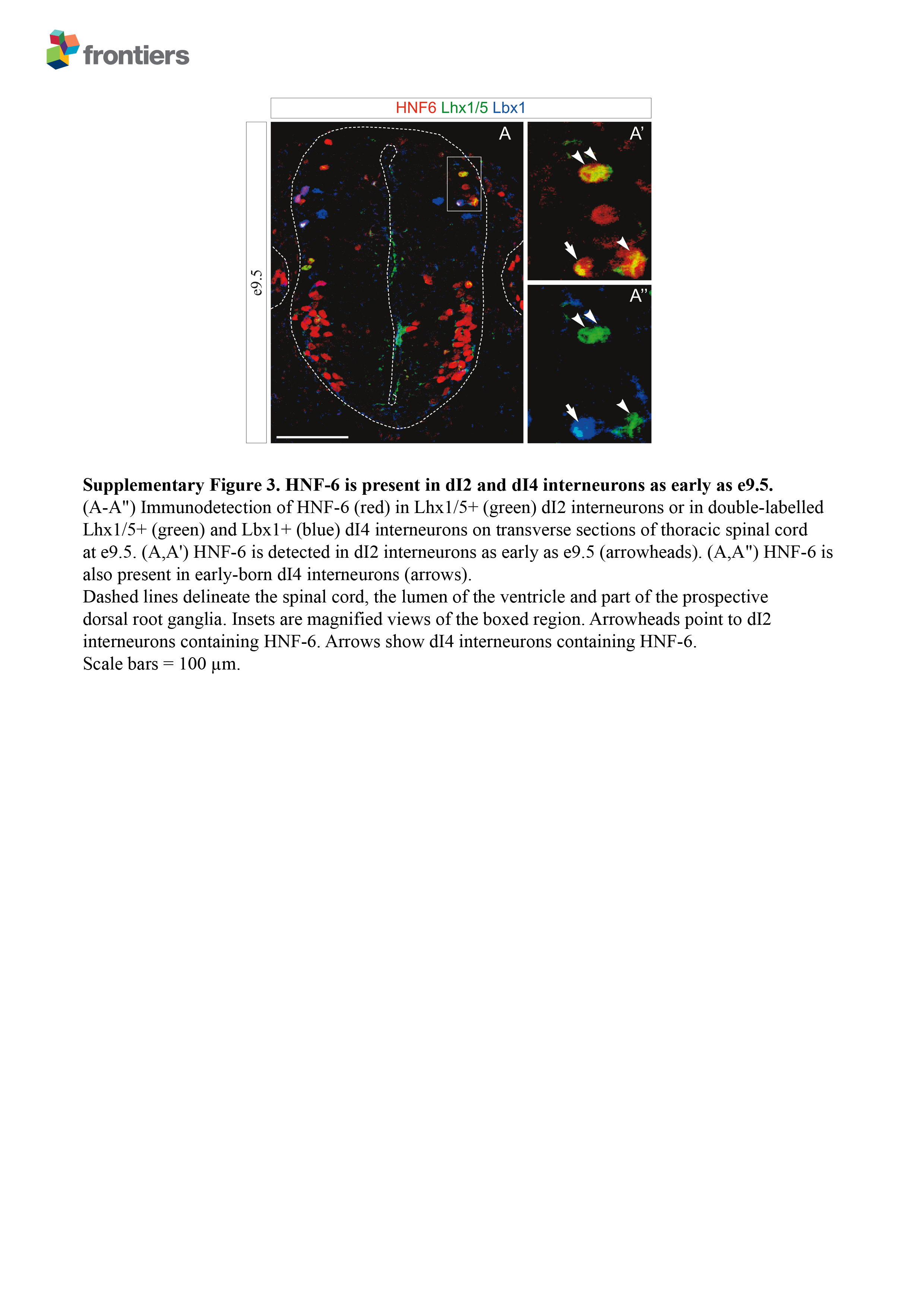

Supplement: Supplementary file 3 [file Image_3.jpeg]

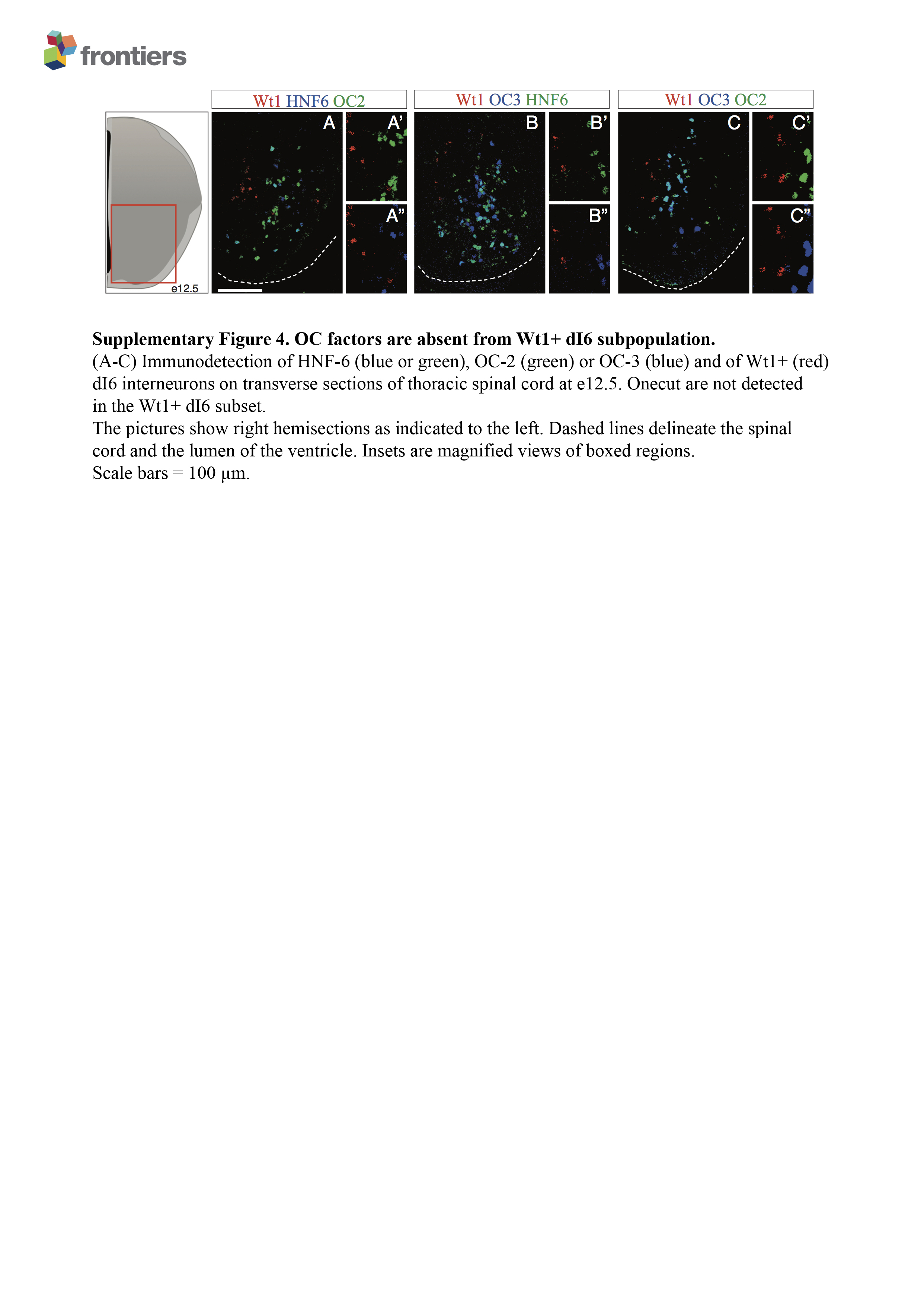

Supplement: Supplementary file 4 [file Image_4.jpeg]
